# Urban Transmission of Human African Trypanosomiasis, Gabon

**DOI:** 10.3201/eid1801.111384

**Published:** 2012-01

**Authors:** Fabrice Simon, Marie Mura, Frédéric Pagès, Gabriel Morand, Philippe Truc, Francis Louis, Philippe Gautret

**Affiliations:** Hôpital d’Instruction des Armées Laveran, Marseille, France (F. Simon, M. Mura, G. Morand, P. Gautret);; Institut de Médecine Tropicale du Service de Santé des Armées, Marseille (F. Pagès);; Institut de Recherche pour le Développement, Montpellier, France (P. Truc);; Organisation de Coordination pour la Lutte contre les Endémies en Afrique Centrale, Yaoundé, Cameroon (F. Louis)

**Keywords:** Sleeping sickness, human African trypanosomiasis, *Trypanosoma brucei gambiense*, urban, transmission, Gabon, *Suggested citation for this article*: Simon F, Mura M, Pagès F, Morand G, Truc P, Louis F, et al. Urban transmission of human African trypanosomiasis, Gabon [letter]. Emerg Infect Dis [serial on the Internet]. 2012 Jan [*date cited*]. http://dx.doi.org/10.3201/eid1801.111384

**To the Editor:** We describe a confirmed case of human African trypanosomiasis (HAT) in an expatriate returning to France from Gabon after a probable tsetse fly bite in the urban setting of Libreville. This case indicates a possible urban transmission of HAT in Gabon and stresses the need for entomologic studies in Libreville.

HAT is endemic to sub-Saharan Africa. *Trypanosoma brucei rhodesiense* (eastern Africa) and *T.b. gambiense* (western Africa) parasites are transmitted to humans by tsetse flies of the *Glossina morsitans* group (*T.b. rhodesiense*) and of the *G. palpalis* group (*T.b. gambiense*), which are found only in Africa. *T.b. gambiense* represents >90% of all reported cases of HAT worldwide. HAT has always been a travel-associated disease. It is a rare cause of fever, cutaneous lesions, and neurologic signs in travelers returning from disease-endemic areas and involves *T.b. rhodesiense* in 70% of the cases, resulting mostly from an exposure during safari in game parks (*1**,**2*).

A 58-year-old previously healthy Portuguese man who worked in Gabon for 13 years for a French company was admitted to the tropical and infectious diseases ward because of a 2-month history of intermittent fever, fatigue, and a 10-kg weight loss. The patient recalled a painful unidentified insect bite on his right thigh 2 months before in his garden in Libreville (Lalala quarter). A 8-cm, indurated, erythematous, and painful plaque (chancre) progressively developed (Figure) in the following weeks after the assumed insect bite. When admitted to the hospital, the patient had a temperature of 39°C, anorexia, insomnia, pruritus of the left arm, and paresthesia of the hands and feet. Two additional large annular erythematous macules, centrally pale (trypanids), were found on his back (Figure). A subclavicular 0.5-cm lymph node was observed. There was no hepatosplenomegaly.

**Figure:**
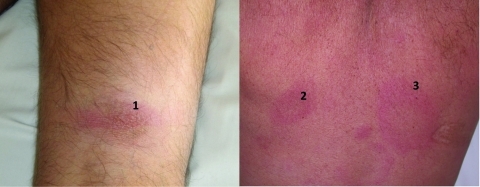
Thigh chancre (1) and back trypanids (2 and 3) in a patient with human African trypanosomiasis infection, Gabon.

His laboratory results showed moderate anemia (hemoglobin 11.8 g/dL) and thrombopenia (134,000 platelets/mm^3^) and elevated levels of C-reactive protein (30.6 mg/L) and gammaglobulins (23.9 g/L). A thick-blood smear showed no malaria parasites but a few trypomastigotes of *Trypanosoma* spp*.* PCR of blood identified *T.b. gambiense.* A cerebrospinal fluid sample showed moderate elevation of total proteins (0.43 g/L) and albumin (291 mg/L), 11 leukocytes, and no IgM elevation. Direct examination and PCR showed no trypanosome in the cerebrospinal fluid. Specific antibodies were found in the blood by indirect immunofluorescence (titer 200). Biopsies of 2 skin lesions (thigh, back) showed a lymphoplasmocytic vasculitis consistent with cutaneous locations of HAT; no parasite was observed in situ. The patient was treated successfully with a 7-day course of pentamidine. The case was reported to World Health Organization Control of Neglected Tropical Diseases Department.

A total of 328 HAT cases were reported to the World Health Organization in Gabon during 2000–2009; most infections were acquired in the mangrove swamp Atlantic coast focus in Noya (Estuaire Province) and some in the focus of Bendje (Ogooué-Maritime Province) (*3*). Four of 6 cases of *T.b. gambiense* imported to Europe during 2005–2009 were in expatriates with a travel history to Gabon (*1*). In the 4 case-patients infected in Gabon, an exposure in rural forest areas was assessed (*4**–**6*; D. Malvy, pers. comm.). In the fifth case reported here, the tsetse bite likely occurred in the urban setting of Libreville.

The patient did not report occupational exposure to tsetse bites outside Libreville during the previous year. He occasionally went in Pointe Denis during weekends but did not remember having been bitten by a tsetse fly. Although the patient did not identify the insect in his garden, the chronology of his clinical history and the presence of a typical chancre at the place of the insect bite that occurred before symptoms provide strong arguments in favor of this hypothesis. The bite occurred during the morning hours, in the patient’s home garden in the Lalala area of Libreville (0.357568N, 9.475365E) near the Ogombié River. This area is located 125 km and 75 km from the Bendje and Noya HAT foci, respectively.

Two studies provided evidence for urban transmission of HAT in Kinshasa (Democratic Republic of Congo) and in Bonon (Côte d’Ivoire) (*7**,**8*). Concurrently, some tsetse species, such as *G. palpalis*, adapt to high human densities and are found in the largest urban centers of western Africa (*9*). Entomologic studies in Libreville should prompt further investigation into a possible urban transmission of HAT in Gabon, as we suspect in the case reported.
